# Refractory Ulcerative Colitis Successfully Treated With a Combination of Granulocyte and Monocyte Apheresis and Mirikizumab: A Case Report

**DOI:** 10.7759/cureus.89747

**Published:** 2025-08-10

**Authors:** Tomotaka Tanaka, Yoshikazu Yoshifuku, Hajime Teshima, Kazuyoshi Nakata, Kosei Kitamura, Nodoka Otabe, Takeshi Mori, Songde Cho, Michihiro Nonaka, Yoshifumi Fujimoto

**Affiliations:** 1 Department of Gastroenterology, Japan Agricultural Cooperatives Hiroshima Kouseiren Hospital, Hiroshima, JPN

**Keywords:** adverse effects of immunomodulators, combination therapy, granulomonocytapheresis, mirikizumab, ulcerative colitis

## Abstract

We report the case of a 42-year-old man with treatment-refractory ulcerative colitis (UC), complicated by steroid dependence, adverse effects of immunomodulators, and significant social limitations, including inability to be hospitalized or take oral medications. Granulocyte and monocyte adsorptive apheresis (GMA) was initiated as an induction therapy, followed by the addition of mirikizumab, an anti-IL-23p19 monoclonal antibody. This sequential combination therapy resulted in significant clinical improvement and endoscopic mucosal healing. A detailed breakdown of the clinical course, objective disease activity markers, and colonoscopic findings is provided to illustrate the rationale and effectiveness of this approach. This case suggests that a stepwise treatment strategy combining GMA and biologics may be effective in managing treatment-refractory UC with complex clinical backgrounds. To our knowledge, this is the first reported case demonstrating the effectiveness of combination therapy with GMA and mirikizumab in UC.

## Introduction

Ulcerative colitis (UC) is a chronic inflammatory bowel disease with a rising global prevalence. When disease control is inadequate, it can significantly impair patients' quality of life due to persistent symptoms and treatment burdens. UC is a chronic inflammatory bowel disease characterized by relapsing and remitting inflammation of the colonic mucosa. The introduction of biologics and small-molecule agents, including tumor necrosis factor-alpha (TNF-α) inhibitors, anti-α4β7 integrin antibodies, IL-12/23 and IL-23p19 inhibitors, and Janus kinase (JAK) inhibitors, has improved treatment outcomes [1-3]. However, therapeutic choices remain difficult in patients with refractory disease or those who cannot tolerate immunosuppressive therapy. In particular, patients with contraindications to hospitalization or oral drug intake present a unique therapeutic challenge, and in this case, it was presumed that the worsening of symptoms led to psychological difficulty in oral intake. Granulocyte and monocyte adsorptive apheresis (GMA) is a non-pharmacologic therapy that selectively removes activated myeloid cells, and is considered safe even in elderly or immunocompromised patients [4]. While effective in inducing clinical response, GMA alone may be insufficient to achieve mucosal healing [5]. The rationale for combining GMA with targeted biologics lies in their complementary mechanisms: GMA acts on early-phase neutrophil-driven inflammation, while biologics such as mirikizumab suppress chronic adaptive immune responses. Mirikizumab, a selective IL-23p19 monoclonal antibody, has shown promising results in inducing and maintaining remission, including endoscopic healing. Here, we report a case of treatment-refractory UC successfully managed with a stepwise combination of GMA and mirikizumab. We obtained the patient's consent for the preparation of this manuscript.

## Case presentation

**Figure 1:**
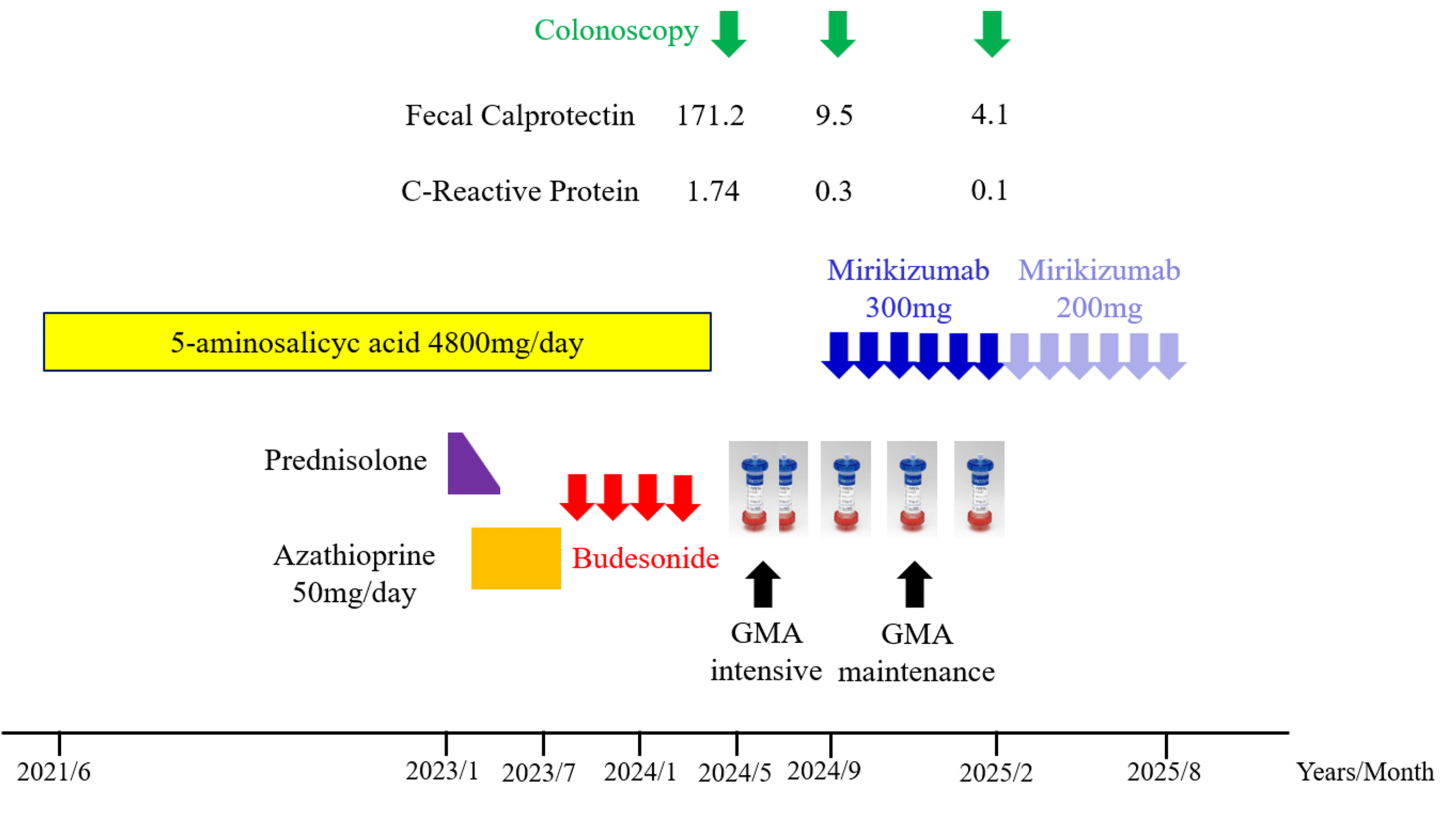
Clinical course. GMA: granulocyte and monocyte adsorptive apheresis.

The patient was a 42-year-old man who was diagnosed with left-sided UC in June 2021. Remission was achieved and maintained with oral 5-aminosalicylic acid (5-ASA) at a dose of 4800 mg/day. However, he experienced a relapse in January 2023, for which prednisolone (30 mg/day) was initiated, leading to remission induction. Nevertheless, due to repeated relapses during steroid tapering, he was considered to have steroid-dependent UC. The addition of azathioprine (AZA) (50 mg/day) enabled successful steroid withdrawal. However, starting in July of the same year, the patient developed numbness in the hands and feet, raising suspicion of peripheral neuropathy, and azathioprine was subsequently discontinued. The neurological symptoms disappeared after discontinuation of AZA. During subsequent relapses, he managed to maintain remission using budesonide foam enemas. However, from January 2024, his symptoms gradually worsened, and by May 2024, he was referred to our hospital due to difficulty with oral intake and food consumption. The clinical course is shown in Figure 1. 

**Figure 2:**
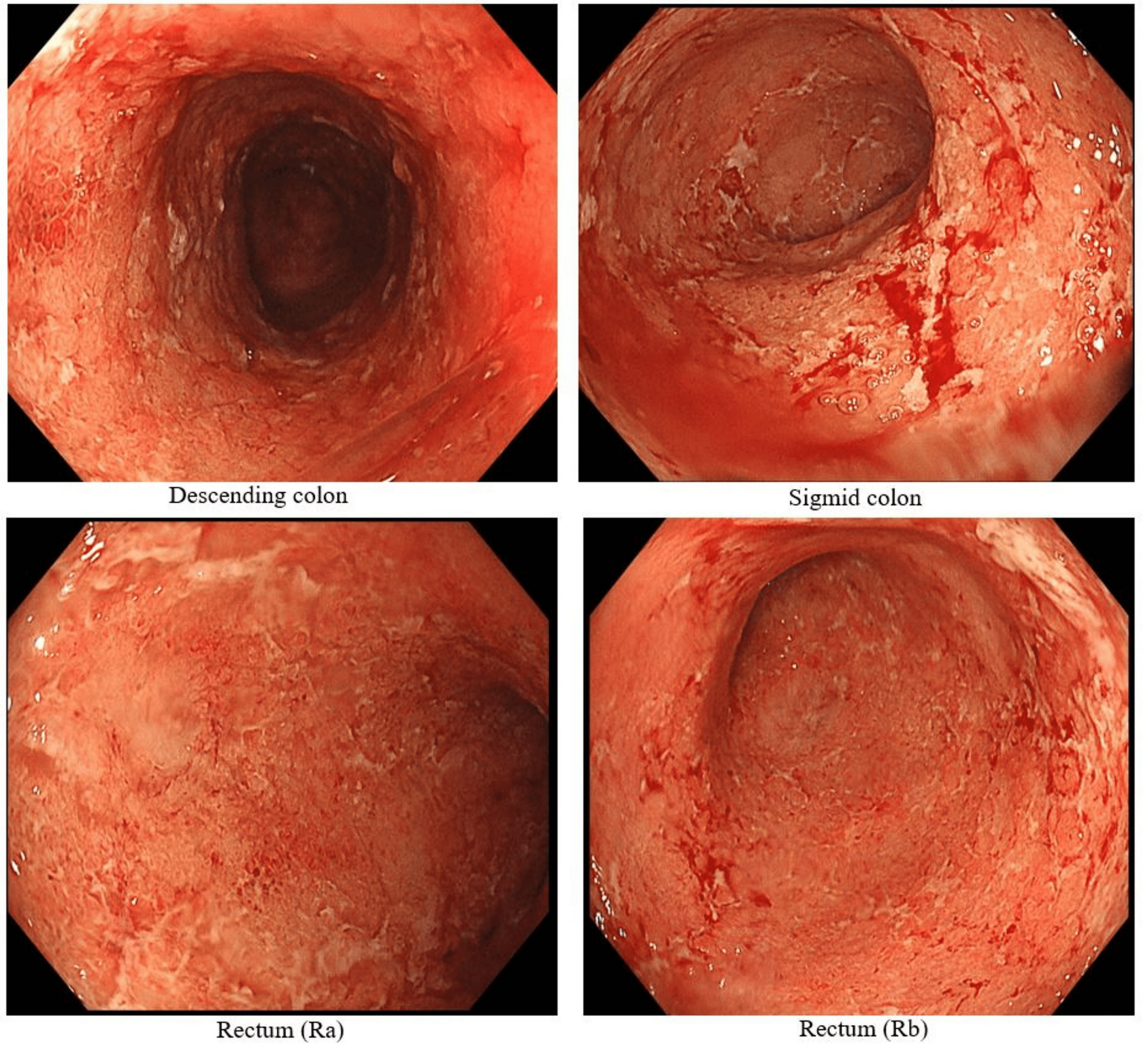
Colonoscopy photo (first visit, May 2024). Persistent erythema, erosions, whitish exudate, and loss of vascular pattern were observed from the rectum to the descending colon.

Blood test data (Table 1) revealed mildly elevated white blood cell, C-reactive protein, and platelet levels, along with mildly decreased total protein, albumin, and cholinesterase levels. Fecal calprotectin was elevated at 171.1 mg/kg. He presented with more than 10 episodes of bloody diarrhea per day and abdominal pain. The Mayo score was 11 points, indicating severe disease. The full Mayo score was 11 (stool frequency = 3, rectal bleeding = 3, endoscopic findings = 3, physician's global assessment = 2), indicating severe disease. Colonoscopy (Figure 2) revealed erythema, erosions, whitish exudate, and loss of vascular pattern from the rectum to the descending colon. Stool culture was positive for *Escherichia coli* (O16), and treatment was initiated with intravenous ceftriaxone and intensive granulocyte and monocyte adsorptive apheresis (GMA) twice weekly as induction therapy for remission. His symptoms gradually improved, and fecal calprotectin decreased to 9.5 mg/kg. GMA was then switched to maintenance therapy twice monthly.

**Table 1:** Blood test and fecal calprotectin. Bold values indicate abnormal laboratory results that fall outside the reference range.

Parameters	Laboratory values	Reference range
White blood cell	10,200/μl	3300-8600
Neutrophil	66.10%	37-73
Lymphocyte	11.00%	20-55
Monocyte	12.90%	2.5-10
Eosinocyte	8.70%	0.5-11
Basophil	1.30%	0-2
Red blood cell	361 x 10^4^/μl	435-555
Hemoglobin	10.0 g/dl	13.7-16.8
Hematocrit	32.5%	40.7-50.1
Platelet	42.0 x 10^4^/μl	15.8-34.8
Total protein	6.3 g/dl	6.5-8.0
Albumin	3.6 g/dl	3.7-5.3
Aspartate aminotransferase	14 U/l	8-38
Alanine aminotransferase	10 U/l	4-44
Cholinesterase	182 U/l	185-431
Lactate dehydrogenase	136 U/l	124-222
Total-bilirubin	0.4 mg/dl	0.2-1.0
Blood urea nitrogen	11 mg/dl	6-24
Creatinine	0.71 mg/dl	0.6-1.1
C-reactive protein	1.74 mg/dl	0.0-0.5
Fecal calprotectin	171.1 mg/dl	0-50

**Figure 3:**
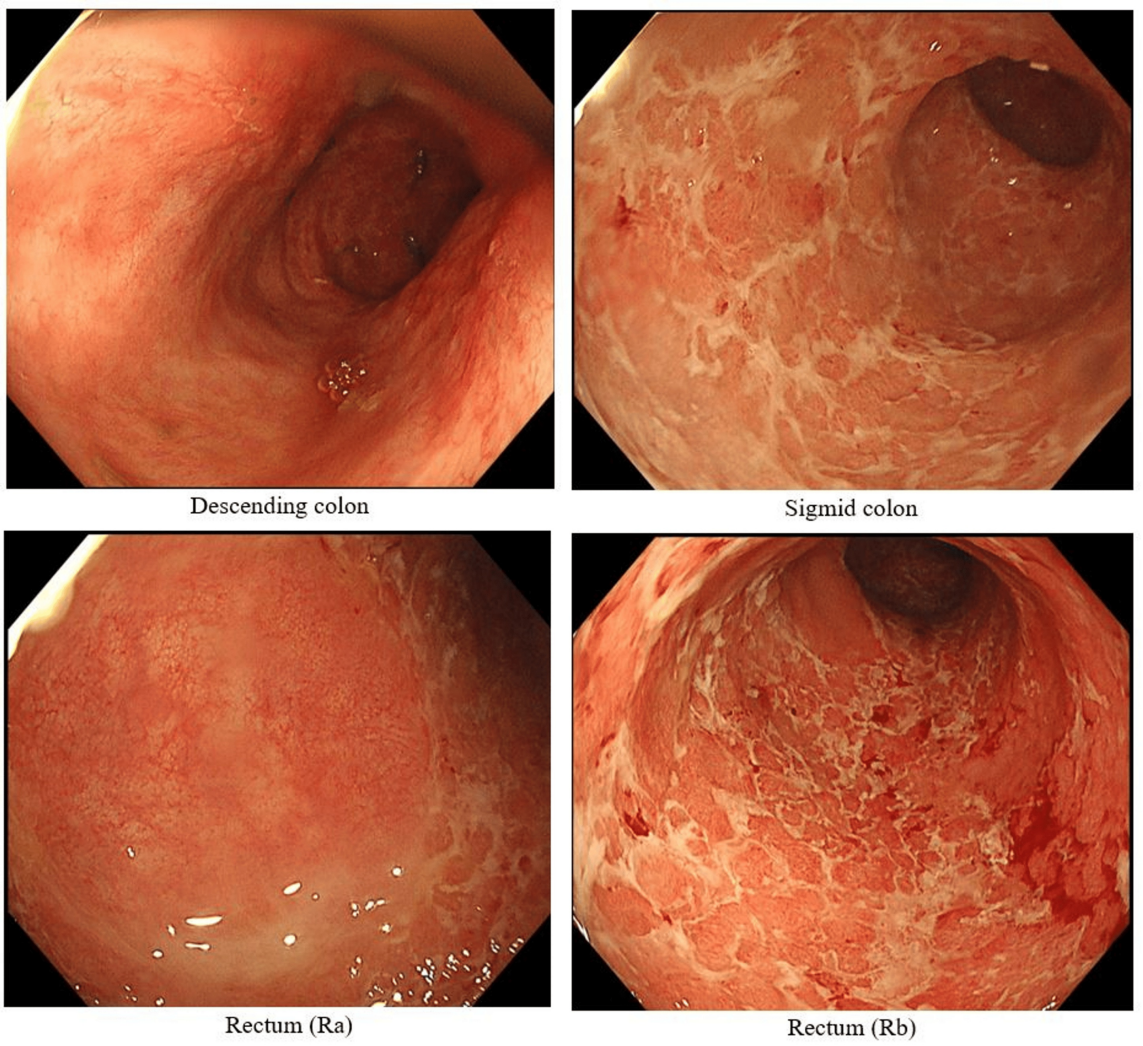
Colonoscopy photo (September 2024, at the end of GMA). Compared to the initial examination, erythema and erosions in the descending colon have improved; however, the sigmoid colon and rectum mucosa still show mucus and loss of vascular pattern. GMA: granulocyte and monocyte adsorptive apheresis.

**Figure 4:**
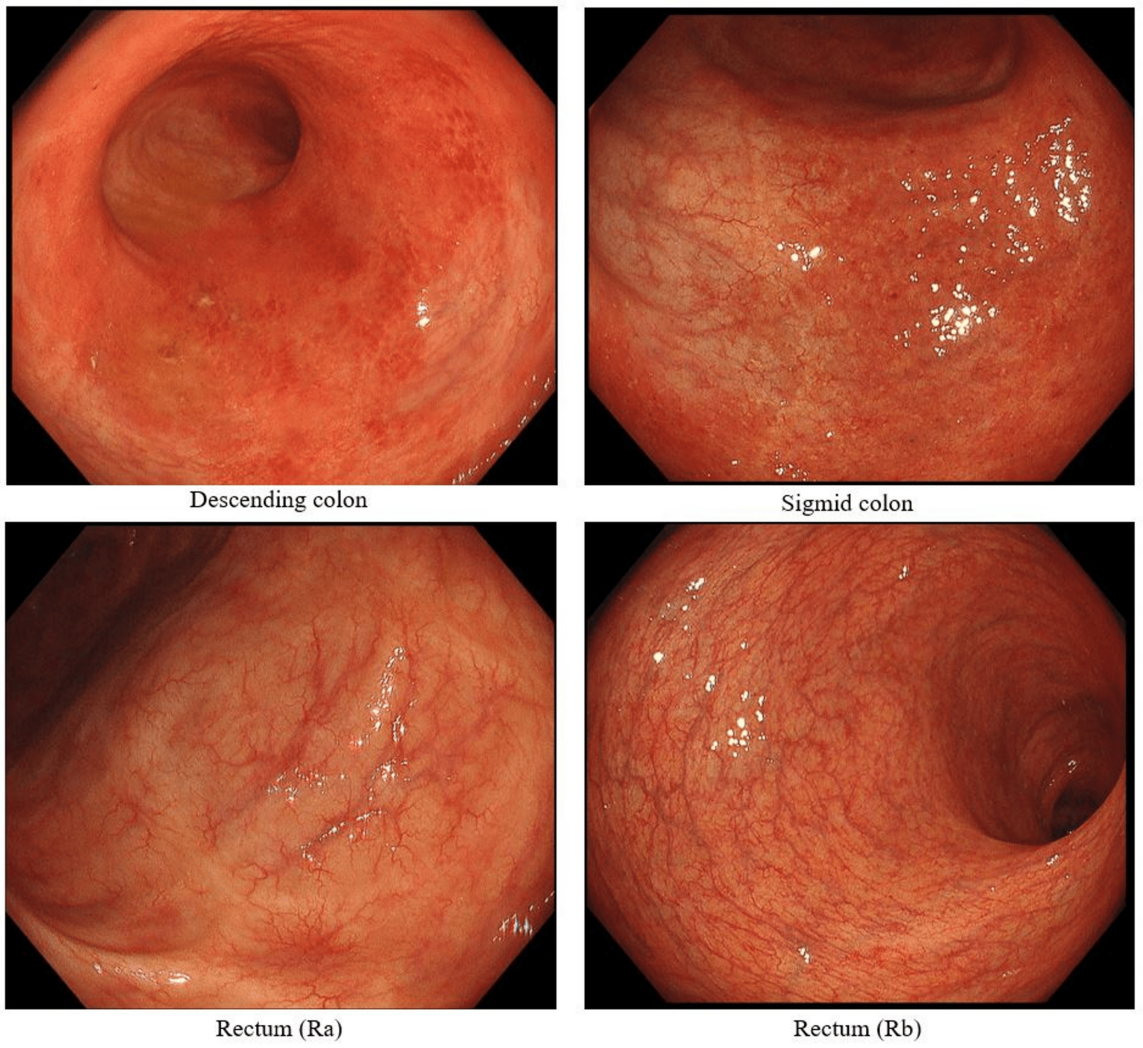
Colonoscopy photo (February 2025, at the end of mirikizumab 300 mg). Vascular pattern of the rectal mucosa has improved, suggesting endoscopic mucosal healing.

In September 2024, a follow-up colonoscopy (Figure 3) showed improvement in erythema and erosions from the descending colon to the rectum. However, whitish exudate persisted in the sigmoid colon and rectum, and the vascular pattern remained absent. Although abdominal pain and hematochezia had improved, the patient continued to experience bowel emergency, interfering with his work. Given the persistent mucosal inflammation despite symptomatic relief, treatment options were discussed with the patient. One of the reasons the patient chose mirikizumab was that maintenance therapy could be administered via self-injection. We initiated combination therapy with intravenous mirikizumab (300 mg) while continuing GMA maintenance therapy. By February 2025, fecal calprotectin had decreased to 4.0 mg/kg, and colonoscopy (Figure 4) confirmed endoscopic mucosal healing. At present, the patient continues maintenance therapy with subcutaneous mirikizumab (200 mg) without relapse.

## Discussion

This case represents a particularly challenging instance of UC, characterized by relapse following remission induction and maintenance with 5-ASA, the development of steroid dependency, intolerance to immunomodulators (azathioprine), inability to take oral medication, ineligibility for hospitalization, and concomitant *Escherichia coli* infection. In such situations, even though potent immunosuppressive therapies such as biologics or JAK inhibitors may be indicated, their initiation and maintenance may not be feasible due to the patient's physical or social circumstances. Therefore, treatment decisions must incorporate not only medical indications but also social appropriateness and patient context [6]. GMA has been reported as a viable bridging therapy under such complex conditions [6]. GMA selectively removes activated neutrophils and monocytes, thus physically suppressing early-phase inflammation. It is well tolerated, associated with minimal adverse effects, and can be administered on an outpatient basis, making it particularly suitable for patients who are unable to be hospitalized or in whom immunosuppressive drugs are contraindicated due to infections. GMA is also gaining attention as a temporal bridging option during treatment transitions or in cases of therapeutic resistance [6].

In this case, GMA led to marked improvement in clinical symptoms such as hematochezia and abdominal pain, along with a substantial reduction in fecal calprotectin. However, colonoscopic findings revealed persistent whitish exudates and loss of vascular pattern in the rectum and sigmoid colon, indicating that mucosal healing had not been achieved. This limitation of GMA in resolving mucosal-level inflammation has been highlighted in several recent studies [7,8].

To address this, mirikizumab, an anti-IL-23p19 monoclonal antibody, was introduced. Mirikizumab targets the chronic inflammatory pathway mediated by Th17 cells and has been shown to have a strong association with mucosal healing [9,10]. The LUCENT-1 and LUCENT-2 trials demonstrated high rates of clinical remission and endoscopic improvement, even in the absence of corticosteroids, positioning mirikizumab as a highly responsive novel biologic [9,10]. In the present case, fecal calprotectin further declined after initiation of mirikizumab, and endoscopic mucosal healing was achieved within six months, suggesting that the combination of GMA and mirikizumab worked synergistically. Among available biologics, mirikizumab was chosen for its steroid-sparing profile, outpatient-friendly administration, and efficacy in moderate-to-severe UC. TNF-α inhibitors and JAK inhibitors were considered but deemed unsuitable due to the patient’s infection history, azathioprine intolerance, and inability to attend inpatient infusion schedules.

In addition, numerous reports have described the combined use of GMA with other biologics or JAK inhibitors in refractory UC. In combination with adalimumab, preceding GMA reduced inflammatory cell burden, followed by TNF-α suppression, achieving clinical remission rates of 55-66% and mucosal healing rates of up to 67% [11]. With ustekinumab, approximately 50% of patients achieved remission, suggesting complementary effects through immunomodulation [12,13]. The combination with vedolizumab is hypothesized to synergize via local inhibition of lymphocyte trafficking and systemic immune modulation by GMA, with several case reports supporting its efficacy [14,15]. JAK inhibitors such as tofacitinib and upadacitinib, when combined with GMA, enable a dual mechanism targeting both intracellular signaling and inflammatory cell depletion, potentially leading to early steroid withdrawal and mucosal healing [16,17]. Golimumab in combination with GMA has shown success in inducing remission in refractory cases where vedolizumab or ustekinumab had failed [18].

Compared with this report, the GMA plus mirikizumab combination in this case demonstrated comparable, if not superior, effectiveness. While GMA relieved abdominal pain and hematochezia, urgency remained; mirikizumab was selected to address this residual symptom burden [9,10]. The stepwise mechanism of GMA, which eliminates peripheral inflammatory cells, and mirikizumab, which inhibits Th17-driven chronic inflammation, represents a phase-targeted and strategic therapeutic approach.

Some clinical insights gained from this case include the following: GMA is effective as an initial outpatient intervention, particularly for patients who are unable to be hospitalized or take oral medications. GMA alone may only achieve symptomatic relief, but combined therapy increases the likelihood of mucosal healing. Mirikizumab, as a novel biologic, is a practical and effective partner in combination therapy. Furthermore, GMA can serve as a flexible bridging strategy, facilitating the initiation or switching of biologics and JAK inhibitors.

Prospective clinical trials investigating the combination of GMA with various agents, patient stratification studies to identify populations most likely to benefit from GMA, and cost-effectiveness analyses will be essential moving forward. Although current evidence is largely based on case reports and small-scale studies, the accumulation of successful examples such as this case may contribute to the diversification and refinement of UC treatment strategies.

This case highlights the effectiveness of a stepwise approach, wherein initial symptom relief was achieved with GMA and subsequent mucosal healing was attained with mirikizumab. Further prospective research will be crucial in establishing practical therapeutic guidelines for combination strategies involving GMA.

A search using PubMed revealed no reports on the combination therapy of granulocyte apheresis and mirikizumab. This report represents, to our knowledge, one of the first documented cases of successful sequential GMA and mirikizumab therapy in a real-world outpatient setting.

## Conclusions

This case illustrates that stepwise combination therapy with GMA followed by mirikizumab can induce both clinical and endoscopic remission in treatment-refractory UC patients with significant therapeutic limitations. This report provides preliminary evidence that sequential therapy with GMA and IL-23p19 inhibition may be a feasible and safe alternative for patients unable to start intensive immunosuppressive treatment. Further research is warranted to determine the optimal timing, patient selection, and long-term outcomes of this combination approach.
